# What Will Happen If We Do Nothing To Control Trachoma: Health Expectancies for Blinding Trachoma in Southern Sudan

**DOI:** 10.1371/journal.pntd.0000396

**Published:** 2009-03-17

**Authors:** Jeremiah M. Ngondi, Fiona E. Matthews, Mark H. Reacher, Jonathan King, Carol Brayne, Hebe Gouda, Paul M. Emerson

**Affiliations:** 1 Department of Public Health and Primary Care, Institute of Public Health, University of Cambridge, Cambridge, United Kingdom; 2 The Carter Center, Atlanta, Georgia, United States of America; 3 MRC Biostatistics Unit, Institute of Public Health, Cambridge, United Kingdom; 4 Health Protection Agency, East of England Regional Epidemiology Unit, Institute of Public Health, Cambridge, United Kingdom; Kilimanjaro Centre for Community Ophthalmology, United Republic of Tanzania

## Abstract

**Background:**

Uncontrolled trachoma is a leading cause of blindness. Current global trachoma burden summary measures are presented as disability adjusted life years but have limitations due to inconsistent methods and inadequate population-based data on trachomatous low vision and blindness. We aimed to describe more completely the burden of blinding trachoma in Southern Sudan using health expectancies.

**Methodology/Principal Findings:**

Age and gender specific trachomatous trichiasis (TT) prevalence was estimated from 11 districts in Southern Sudan. The distribution of visual acuity (VA) in persons with TT was recorded in one district. Sudan life tables, TT prevalence, and VA were used to calculate Trichiasis Free Life Expectancy (TTFLE) and Trichiasis Life Expectancy (TTLE) using the Sullivan method. TTLE was broken down by VA to derive TTLE with normal vision, TTLE with low vision, and TTLE with blindness. Total life expectancy at birth in 2001 was 54.2 years for males and 58.1 for females. From our Sullivan models, trichiasis life expectancy at the age of 5 years was estimated to be 7.0 (95% confidence interval [CI] = 6.2–7.8) years (12% [95% CI = 11–14] of remaining life) for males and 10.9 (95% CI = 9.9–11.9 ) years (18% [95% CI = 16–20] of remaining life) for females. Trichiasis life expectancy with low vision or blindness was 5.1 (95% CI = 3.9–6.4) years (9% [95% CI = 7–11] of remaining life) and 7.6 (95% CI = 6.0–9.1) years (12% [95% CI = 10–15] of remaining life) for males and females, respectively. Women were predicted to live longer and spend a greater proportion of their lives with disabling trichiasis, low vision, and blindness compared to men.

**Conclusions:**

The study shows the future burden associated with doing nothing to control trachoma in Southern Sudan, that is, a substantial proportion of remaining life expectancy spent with trichiasis and low vision or blindness for both men and women, with a disproportionate burden falling on women.

## Author Summary

Summary measures of population health attempt to express disease burden in terms of a common “currency” and are useful in establishing public health priorities. Disability adjusted life years (DALYs), a health gap measure, have previously been used to estimate burden due to trachoma; however, their methods and results have limitations. This study demonstrates the application of the health expectancies to estimate burden due to trachoma. The study illustrates the future burden associated with doing nothing to control trachoma in Southern Sudan: a substantial proportion of remaining life expectancy spent with trichiasis and low vision or blindness for both men and women, with a disproportionate burden falling on women. The results presented are intuitively meaningful for policy makers and a non-technical audience and compare favourably with other indicators such as mortality and incidence rates or DALYs, which are not generally easily understood. Unless action is taken by further delivery of trachoma control interventions, then populations in Southern Sudan can expect to spend a substantial proportion of their life with low vision or blindness due to trachoma.

## Introduction

Trachoma is one of the oldest infectious diseases known to mankind and is the leading infectious cause of blindness, estimated to be responsible for 2.9% of blindness worldwide [1]. Recurrent infection with ocular *Chlamydia trachomatis* results in chronic inflammation, scarring, trichiasis, corneal opacification, and blindness [2]–[4]. Blindness due to trachoma is preventable through the World Health Organization (WHO) SAFE strategy which comprises: Surgery, eyelid surgery to correct in-turned eyelashes which stops pain and minimizes risk of corneal damage; Antibiotic treatment for active trachoma using single-dose oral azithromycin or topical tetracycline; Facial cleanliness, promotion of clean faces especially in children through sustained behaviour change; and Environmental improvements to increase access to water and sanitation [5].

Summary measures of population health, including disability adjusted life years (DALYs) [6] and handicap adjusted life years (HALYs) [7], have been used to estimate the global burden attributable to trachoma. DALYs and HALYs are population health measures permitting morbidity and mortality to be simultaneously described within a single number and estimate the gap between a population's health and some defined goal. The methodology and data sources describing trachoma DALYs and HALYs have differed such that direct comparisons are not possible. For instance, Evans and Ranson estimated global burden of trachoma for the year 1990 to be 80.0 million HALYs [7]; while for the same years the Global Burden of Disease (GBD 1990) project reported trachoma burden to be 1.0 million DALYs [8]. Additionally, studies describing the global burden of trachoma for the year 2000 yielded different estimates of 2.2 million DALYs [6] and 3.6 million DALYs [9]. These previous estimates also have limitations arising from paucity of population-based data on trachomatous low vision and blindness [7],[9],[10].

We aimed to demonstrate the application of the health expectancies approach for trachomatous trichiasis health states (any trichiasis, trichiasis with normal vision, trichiasis with low vision, and trichiasis with blindness) as a summary measure of trachoma burden using population-based survey data from Southern Sudan. Health expectancy is a measure that combines information on both mortality and morbidity to derive lengths of time spent in different states of health. The methods presented can be applied to other trachoma endemic areas and presents estimates of the potential burden of blinding trachoma if control measures are not implemented.

## Methods

### Ethics Statement

The Institutional Review Board of Emory University approved the survey protocols and clearance to conduct surveys was obtained from the Sudan Peoples Liberation Movement Secretariat of Health (SPLM/Health). Verbal informed consent to participate was sought from the heads of the household, from each individual and the parents of children aged less than 10 years in accordance with the declaration of Helsinki. Consent for household interviews and eye examination was documented by interviewers and examiners on the data collection forms. Personal identifiers were removed from the data set before analyses were undertaken.

### Cross-Sectional Surveys

Surveys for trachoma were conducted in eleven districts in Southern Sudan between 2001 and 2006 [11]–[13]. For each district, the sample size was calculated to allow for estimation of at least 50% prevalence of active trachoma signs in children aged 1–9 years within a precision of 10% given a 95% confidence limit and a design effect of 5. We also aimed to estimate at least 2.5% prevalence of trachoma trichiasis (TT) in persons aged 15 years and above within a precision of 1.5% at 95% confidence limit and a design effect of 2. The districts were selected on the basis of pragmatic program implementation criteria of: 1) anecdotal reports of blinding trachoma; 2) security and accessibility; and 3) feasibility of initiating trachoma control interventions after the survey.

A two-stage cluster random sampling with probability proportional to size was used to select the sample population in each district. A cluster was defined as the population within a single village. Using a line listing of all the villages in each survey district, villages were grouped into sub-districts. Villages that were inaccessible and/or insecure were excluded from the sampling frame. In the first stage, villages were randomly selected with probability proportional to the estimated population of the sub-district. In the second stage, households were selected from the villages selected in the previous stage using the random-walk method [14], except in Ayod district where the compact segment method [15] was used for sampling households. All residents of selected households were enumerated and those present were eligible for eye examination. It was not possible to return later to the households to pick up any absentees and households where residents were not available were skipped.

### Trachoma Examination

Trainee examiners comprising of auxiliary nurses and community health workers were trained using the WHO simplified grading system [16] by a senior examiner experienced in trachoma grading (ophthalmologist or ophthalmic nurse). The minimum accepted inter-observer agreement was set at 80% and reliability assessed in two stages. In the first stage, trainee examiners identified trachoma grades using the WHO sets of trachoma slides [17],[18]. Those examiners who achieved at least 80% agreement then proceeded to the second stage of field evaluation. During field evaluation a reliability study comprising 50 persons of varying age and gender were selected by the ophthalmic nurse to represent all trachoma grades. Each trainee examiner evaluated all 50 subjects independently and recorded their findings on a pre-printed form. Inter-observer agreement was then calculated for each trainee using the senior examiners' observation as the ‘gold standard’. Only trainees achieving at least 80% inter-observer agreement after the field evaluation were included as trachoma graders.

All persons living within each selected household who gave verbal consent were examined using a torch and a ×2.5 magnifying binocular loupe in accordance to the simplified grading system. Alcohol-soaked cotton-swabs were used to clean the examiner's fingers between examinations. All examined participants were assigned a dichotomous outcome for each trachoma sign based on the worst affected eye. TT was defined by the presence of at least one eye lash touching the eyeball or evidence of epilation of the eyelashes. Individuals with signs of active trachoma were offered treatment with 1% tetracycline eye ointment. Patients TT were referred to the health centre where free eyelid surgery was available.

### Visual Acuity Testing

In one district (Mankien), visual acuity (VA) testing was conducted in all eligible participants [19]. Experienced Integrated eye care workers (IECW) were re-trained in VA testing, basic eye examination and trachoma grading and their reliability assessed. Only trainees achieving an inter-observer agreement of 80% and above were eligible to participate as examiners. Prior to the survey, the minimum age for visual acuity (VA) testing was predetermined to be 5 years. VA testing was conducted outdoors in adequate sunlight using the Snellen E chart at 6 meters. In persons with VA<6/60, VA was evaluated with the Snellen chart at 3 meters. Further VA assessment was done in persons with VA<3/60 by counting fingers, hand movement and light perception as appropriate. All participants then underwent basic eye examination. Using a torch and a ×2.5 magnifying binocular loupe, each eye was examined first for in-turned lashes (TT), and the cornea was then inspected for corneal opacities (CO), and the lens examined for cataract. Persons with visual impairment were referred to attend an eye surgery-camp conducted after the survey.

Data were recorded on a customized form and the cause of visual impairment determined for all subjects with a presenting VA of <6/18 for each eye separately. The principal disorder responsible for low vision or blindness was determined for the participant by taking into account the main cause for each individual eye. Vision loss was attributed to trachoma in persons presenting with trichiasis and corneal opacity. In the instance where different causes of vision loss had been identified for each eye separately in a given individual, the principal disorder was chosen to be the one that was most readily curable or, if not curable, most easily preventable (i.e. cataract, trachoma, non-trachomatous CO, and other causes in that order). To define the vision status we adopted the WHO categories of visual impairment based on presenting visual acuity (Box 1).

**Box 1. Definitions of vision status based on presenting visual acuity: adopted from international statistical classification of diseases and related health problems (ICD-10; Block H53-H54) [34]**

- *Blindness*: presenting visual acuity of less than 3/60 in the better eye
- *Low vision*: presenting visual acuity of less than 6/18 but equal to or better than 3/60 in the better eye
- *Normal vision*: presenting visual acuity of better than or equal to 6/18 in the better eye
- *Visual impairment*: refers to presence of either low vision or blindness

### Distribution of Vision Status

Our model of the distribution of vision status has been described previously [19]. In brief; using VA data for persons presenting with TT from Mankien survey, age specific distributions of vision status were calculated for 5-year age intervals. We then fitted an ordinal logistic regression model to the observed data to explore the age and gender distribution of the three categories of vision status: normal vision; low vision; and blindness. Persons with visual impairment not directly attributable to trichiasis were excluded from the final model. Children aged 0–4 years were assumed to have normal vision. Predicted probabilities were derived to smooth age-specific curves for the three categories of vision status.

### Life Tables for Sudan

Life tables are frequently used in demography, actuarial science and health services. They trace the life expectancy in pre-determined intervals for a hypothetical population size (frequently 100,000 births) based on parameters usually derived from vital registration data. Abridged life tables for Sudan for the year 2001 were obtained from the World Health Organisation Statistical Information System (WHOSIS) for males and females separately [20]. Demographic estimates for Sudan are based on model life tables because vital registration data are poor or not available. The life tables were derived using the Modified Logit model life table system which is extensively used for countries with poor vital registration. The Modified Logit system has been modelled using data from other populations judged to be similar and is indexed on the number of survivors at age five years and the number of survivors at age 60 years [21].

### Definition of Health States

The health states used to describe the burden due to trachoma were defined as follows: Total Life Expectancy, the total lifespan at birth (years); Trichiasis Free Life Expectancy (TTFLE), the expectation of life without any trichiasis; and Trichiasis Life Expectancy (TTLE), the expectation of life with any trichiasis. TTLE was then broken down into three health states: 1) TTLE with normal vision, the expectation of life with any trichiasis and normal vision (presenting VA≥6/18 in better eye); 2) TTLE with low vision, the expectation of life with any trichiasis and low vision (presenting VA<6/18 but ≥3/60 in better eye); and 3) TTLE with blindness, the expectation of life with any trichiasis and blindness (presenting VA<3/60 in the better eye).

### Statistical Analysis

**Figure 1:**
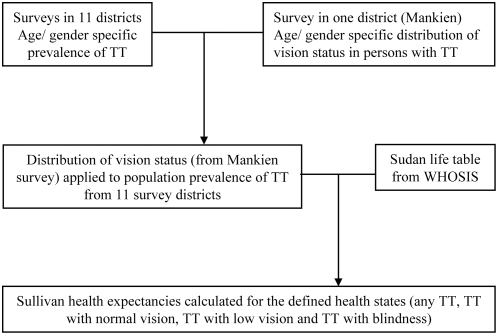
Summary of data framework for analysis of trichiasis health expectancies. TT, trachomatous trichiasis; WHOSIS, World Health Organization Statistical Information System.

The data analysis framework is summarised in Figure 1. Microsoft Excel spreadsheets developed by the European Health Expectancy Monitoring Unit were adapted for the calculation of health expectancies [22]. Age and gender specific prevalence of trichiasis was estimated from cross-sectional surveys and modelled using logistic regression to smooth the prevalence estimates. The distribution of vision status was derived from Mankien survey whereby visual acuity was categorized into normal vision, low vision and blindness; and modelled by ordinal logistic regression to provide proportions for each category of VA by age and gender [19]. The prevalence of vision status in the sample population was then calculated by multiplying the age and gender specific proportions of vision status with the smoothed prevalence of trichiasis. Life tables were collapsed to represent 5-year age-groups from age zero (0) to 75 years and above. Trichiasis Free Life Expectancy (TTFLE) and Trichiasis Life Expectancy (TTLE) were then calculated using the Sullivan method [23]. The Sullivan method combines use of life tables and age specific prevalence of morbidity to partition life expectancy into years with and without morbidity. The Sullivan health expectancy reflects the current health of the population adjusted for mortality levels and independent of age structure. Health expectancy calculated by the Sullivan method is the number of remaining years, at a particular age, that an individual can expect to live in a specified health state. Trichiasis Life Expectancy was further broken down by vision status to derive TTLE with normal vision, TTLE with low vision and TTLE with blindness.

## Results

### The Study Population, Prevalence of Trichiasis, and Trichiasis Vision Status

Table 1 summarises the study population. A total of 23,139 (87.2% of those enumerated) people, in 11 districts, were examined for trachoma of whom males comprised 43%. The overall prevalence of trachomatous trichiasis (all ages) was 6.0% (95% confidence interval [CI] = 5.2–7.0) and varied by district ranging from 0.7% in Katigiri to 10.0% in Kimotong. Of 341 people with TT in Mankien district, 319 were included in modelling of the distribution of vision status (i.e. TT with normal vision, TT with low vision and TT with blindness). The distribution of proportions of vision status by age and gender in persons with trichiasis is shown in Table 2 [19].

**Table 1:** The study population and distribution of vision status.

District	Survey Year	Sample Population				Trachomatous Trichiasis	
		People Enumerated	People Examined	Response Rate (%)	People Examined % Males	Number of Cases	Prevalence % (95%CI)
Paluer	2002	3,650	2,999	82.2	39.9	162	5.4 (4.2–6.9)
Padak	2002	2,277	1,822	80.0	46.8	87	4.8 (3.0–7.5)
Kongor	2004	2,475	1,927	77.9	45.3	52	2.7 (1.6–4.7)
Boma	2003	2,576	2,391	92.8	45.5	174	7.3 (3.6–14.1)
Kiechkuon	2002	1,738	1,530	88.0	37.9	125	8.2 (6.5–10.3)
Mankien	2005	3,976	3,567	89.7	44.5	341	9.6 (70–13.0)
Katigiri	2001	1,743	1,642	94.2	43.5	12	0.7 (0.2–2.3)
Tali	2001	1,530	1,433	93.7	42.6	32	2.2 (1.3–3.4)
Narus	2004	2,049	1,681	82.0	35.1	51	3.0 (2.1–4.3)
Kimotong	2004	1,735	1,586	91.4	40.1	159	10.0 (5.1–18.8)
Ayod	2006	2,605	2,561	98.3	48.0	182	7.1 (5.8–10.8)
Total		26,354	23,139	87.8	43.0	1,377	6.0 (5.2–7.0)

**Table 2:** Distribution of predicted proportions of vision status by age group and gender in persons with trichiasis in Mankien district (n = 319).

Age Group (years)	Vision Status** (%)					
	Males			Females		
	Normal Vision	Low Vision	Blindness	Normal Vision	Low Vision	Blindness
0–4*	100.0	0	0	100.0	0	0
5–9	94.6	5.0	0.4	96.3	3.3	0.3
10–14	90.1	9.2	0.7	93.1	6.2	0.7
15–19	83.9	14.8	1.3	88.4	10.4	1.2
20–24	76.1	21.8	2.1	82.2	15.8	2.0
25–29	67.2	29.7	3.2	74.7	22.3	3.0
30–34	57.6	37.7	4.7	66.0	29.4	4.5
35–39	48.2	45.1	6.7	56.9	36.6	6.5
40–44	39.4	51.3	9.3	47.9	43.0	9.1
45–49	31.7	55.7	12.6	39.4	48.2	12.4
50–54	25.2	58.2	16.5	32.0	51.6	16.4
55–59	19.9	58.9	21.2	25.6	53.3	21.1
60–64	15.6	57.9	26.5	20.3	53.1	26.6
65–69	12.2	55.4	32.4	16.0	51.4	32.5
70–74	9.6	51.8	38.6	12.6	48.4	38.9
75+	7.5	47.4	45.0	10.0	44.6	45.5

*: Children aged 0–4 years assumed to have normal vision.

**: Vision status: Normal vision = presenting visual acuity of ≥6/18 in the better eye; Low vision = presenting visual acuity of <6/18 to ≤3/60 in the better eye; Blindness = presenting visual acuity of <3/60 in the better eye.

Table 3 summarises the age and gender specific prevalence of trichiasis and breakdown of prevalence of trichiasis vision status. The prevalence of TT increased with age and females were more likely to have TT compared to males, age adjusted Odds Ratio (OR) = 1.5 (95% CI = 1.3–1.7). Consistent with prevalence of trichiasis, prevalence of visual impairment (low vision and blindness) increased with age (Table 3).

**Table 3:** Age and gender specific prevalence of trichiasis and trichiasis vision status.

Age Group (years)	Males							Females						
	Number of People Surveyed	People with TT	TT Prevalence (Estimated)	TT Prevalence (Smoothed)*	Prevalence of Vision Status**			Number of People Surveyed	People with TT	TT Prevalence (Estimated)	TT Prevalence (Smoothed)*	Prevalence of Vision Status**		
					Normal Vision	Low Vision	Blindness					Normal Vision	Low Vision	Blindness
0–4	2,654	2	0.1%	1.0%	1.0%	0.0%	0.0%	2,504	7	0.3%	1.5%	1.3%	0.0%	0.0%
5–9	2,204	34	1.5%	1.3%	1.3%	0.1%	0.0%	2,316	45	1.9%	2.0%	1.7%	0.1%	0.0%
10–14	1,200	32	2.7%	1.8%	1.6%	0.2%	0.0%	1,198	47	3.9%	2.7%	2.3%	0.2%	0.0%
15–19	653	24	3.7%	2.5%	2.1%	0.4%	0.0%	1,026	41	4.0%	3.6%	2.9%	0.3%	0.0%
20–24	449	29	6.5%	3.3%	2.5%	0.7%	0.1%	1,110	43	3.9%	4.9%	3.7%	0.7%	0.1%
25–29	515	24	4.7%	4.5%	3.0%	1.3%	0.1%	1,229	77	6.3%	6.5%	4.5%	1.4%	0.2%
30–34	448	25	5.6%	6.0%	3.5%	2.3%	0.3%	1,006	78	7.8%	8.6%	5.4%	2.4%	0.4%
35–39	469	50	10.7%	8.0%	3.8%	3.6%	0.5%	806	89	11.0%	11.4%	6.2%	4.0%	0.7%
40–44	345	45	13.0%	10.5%	4.2%	5.4%	1.0%	573	86	15.0%	14.9%	6.9%	6.2%	1.3%
45–49	305	39	12.8%	13.8%	4.4%	7.7%	1.7%	481	115	23.9%	19.2%	7.4%	9.0%	2.3%
50–54	330	48	14.5%	17.9%	4.5%	10.4%	3.0%	426	131	30.8%	24.5%	7.6%	12.3%	3.9%
55–59	128	23	18.0%	22.9%	4.6%	13.5%	4.9%	145	45	31.0%	30.6%	7.7%	16.0%	6.4%
60–64	139	39	28.1%	28.8%	4.5%	16.6%	7.6%	220	85	38.6%	37.5%	7.5%	19.7%	9.9%
65–69	76	13	17.1%	35.5%	4.3%	19.6%	11.5%	70	21	30.0%	44.9%	7.2%	23.0%	14.6%
70–74	31	7	22.6%	42.8%	4.1%	22.1%	16.5%	49	16	32.7%	52.6%	6.7%	25.5%	20.5%
75+	14	7	50.0%	50.4%	3.8%	23.9%	22.7%	20	10	50.0%	60.1%	6.0%	26.9%	27.5%
Total	9,960	441						13,179	936					

TT, trachomatous trichiasis.

*: TT prevalence smoothed by fitting logistic regression curve to estimated TT prevalence.

**: Vision status: Normal vision = presenting visual acuity of ≥6/18 in the better eye; Low vision = presenting visual acuity of <6/18 to ≥3/60 in the better eye; Blindness = presenting visual acuity of <3/60 in the better eye. Prevalence of vision status = smoothed TT prevalence×predicted probability of vision status derived from reference 14.

### Life Table Characteristics for Sudan

In the 2001 Sudan life table, women had a higher life expectancy at birth than men. The life expectancy at birth for Sudan was 58.1 years for females and 54.1 years for males. Life expectancy increased in age 5–9 years compared to life expectancy at birth (age 0–4 years) to 56.8 years in males and 60.7 for females; indicating the high under-five mortality rate.

### Trichiasis Health Expectancies

**Figure 2:**
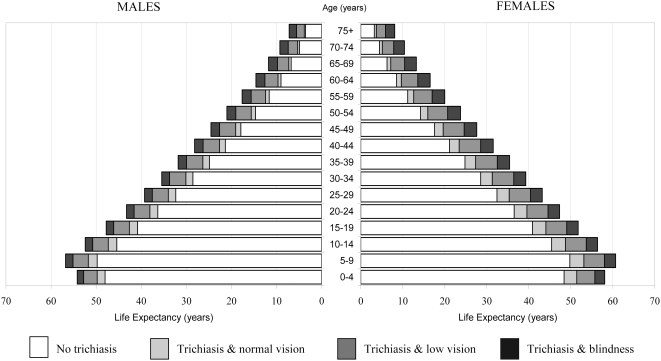
Trichiasis free life expectancy (TTFLE), Trichiasis life expectancy (TTLE) with normal vision, TTLE with low vision and TTLE with blindness by gender. Normal vision = presenting visual acuity of ≥6/18 in the better eye; Low vision = presenting visual acuity of <6/18 to ≥3/60 in the better eye; Blindness = presenting visual acuity of <3/60 in the better eye.

**Figure 3:**
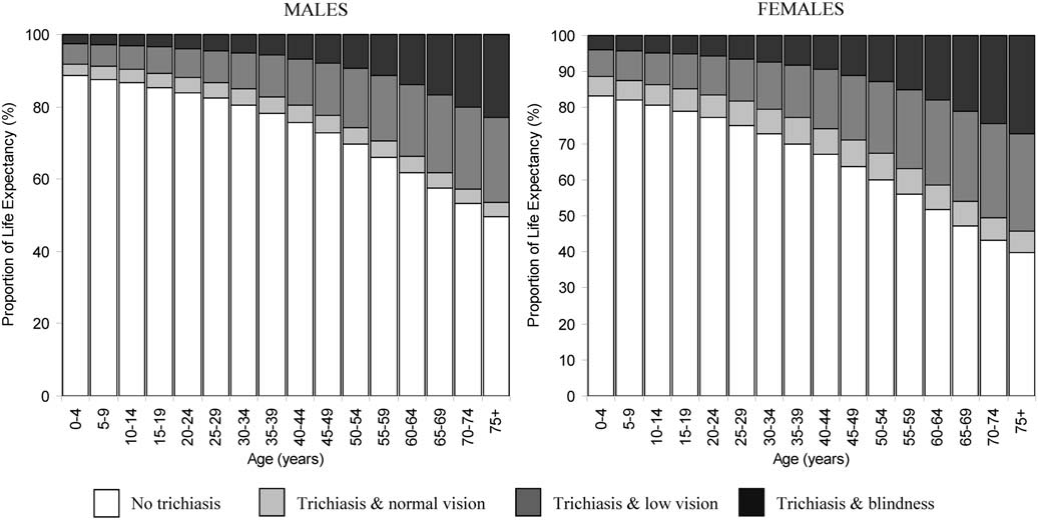
Proportion of life expectancy: without trichiasis; with trichiasis and with normal vision; with trichiasis and low vision; and with trichiasis and blindness by gender. Normal vision = presenting visual acuity of ≥6/18 in the better eye; Low vision = presenting visual acuity of <6/18 to ≥3/60 in the better eye; Blindness = presenting visual acuity of <3/60 in the better eye.

Table 4, Figure 2 and Figure 3 show the life expectancy (LE) and proportions of total life expectancy, trichiasis free life expectancy (TTFLE), trichiasis life expectancy (TTLE), and TTLE with normal vision, TTLE with low vision, and TTLE with blindness. Females had a greater life expectancy at all ages (Figure 2) than males and a larger proportion of remaining life spent with trichiasis low vision or blindness (Figure 3). At age five, TTFLE was 49.8 years (88% of remaining life) and 49.8 years (82% of remaining life) in males and females, respectively. Males expected to live 7.0 (95%CI = 6.2–7.8) years (12% [95% CI = 11–14] of remaining life) with trichiasis at age five; of which 1.9 years (3% of remaining life), 3.5 years (6% of remaining life) and 1.6 years (3% of remaining life) would be lived with normal vision, low vision and blindness, respectively. At age five, females TTLE was 10.9 (95%CI = 9.9–11.9) years (18% [95% CI = 16–20] of remaining life) of which trichiasis with normal vision, low vision and blindness comprised 3.3 years (6%), 4.9 years (8%) and 2.7 years (4%), respectively. For both genders, the proportion of life spent with trichiasis increased with age (Figure 3), by age 50 years, TTLE was 30% (6.4 years) for males and 40% (9.5 years) for females. The proportion of remaining life with trachoma visual impairment (low vision or blindness) at age 50 was 26% (5.5 years) for males and 33% (7.7 years) for females.

**Table 4:** Life expectancy (LE), Trichiasis free life expectancy (TTFLE), Trichiasis life expectancy (TTLE), TTLE with normal vision, TTLE with low vision and TTLE with blindness.

Age Group (years)	Males						Females					
	Life Expectancy (LE)	Trichiasis Free Life Expectancy (TTFLE)	Trichiasis Life Expectancy (TTLE)	TTLE with Normal Vision	TTLE with Low Vision	TTLE with Blindness	Life Expectancy (LE)	Trichiasis Free Life Expectancy (TTFLE)	Trichiasis Life Expectancy (TTLE)	TTLE with Normal Vision	TTLE with Low Vision	TTLE with Blindness
	Years	Years (%)	Years (%)	Years (%)	Years (%)	Years (%)	years	Years (%)	Years (%)	Years (%)	Years (%)	Years (%)
0–4	54.2	48.0 (89)	6.2 (11)	1.7 (3)	3.1 (6)	1.4 (3)	58.1	48.4 (83)	9.7 (17)	3.0 (5)	4.3 (7)	2.4 (4)
5–9	56.8	49.8 (88)	7.0 (12)	1.9 (3)	3.5 (6)	1.6 (3)	60.7	49.8 (82)	10.9 (18)	3.3 (6)	4.9 (8)	2.7 (4)
10–14	52.4	45.4 (87)	7.0 (13)	1.8 (3)	3.5 (7)	1.7 (3)	56.4	45.4 (81)	10.9 (19)	3.3 (6)	4.9 (9)	2.7 (5)
15–19	47.8	40.8 (85)	7.0 (15)	1.7 (4)	3.6 (7)	1.7 (4)	51.8	40.9 (79)	10.9 (21)	3.2 (6)	5.0 (10)	2.7 (5)
20–24	43.3	36.3 (84)	6.9 (16)	1.7 (4)	3.6 (8)	1.7 (4)	47.3	36.5 (77)	10.8 (23)	3.1 (6)	5.0 (11)	2.7 (6)
25–29	39.3	32.3 (82)	6.9 (18)	1.6 (4)	3.6 (9)	1.7 (4)	43.2	32.4 (75)	10.8 (25)	2.9 (7)	5.1 (12)	2.8 (6)
30–34	35.5	28.5 (80)	6.9 (20)	1.5 (4)	3.7 (10)	1.8 (5)	39.3	28.6 (73)	10.7 (27)	2.7 (7)	5.1 (13)	2.9 (7)
35–39	31.8	24.9 (78)	6.9 (22)	1.4 (4)	3.7 (12)	1.8 (6)	35.4	24.8 (70)	10.6 (30)	2.5 (7)	5.2 (15)	2.9 (8)
40–44	28.2	21.4 (76)	6.8 (24)	1.2 (4)	3.7 (13)	1.9 (7)	31.5	21.1 (67)	10.4 (33)	2.3 (7)	5.1 (16)	3.0 (9)
45–49	24.6	17.9 (73)	6.7 (27)	1.1 (4)	3.6 (15)	1.9 (8)	27.6	17.6 (64)	10.0 (36)	2.0 (7)	5.0 (18)	3.0 (11)
50–54	21.0	14.6 (70)	6.4 (30)	0.9 (4)	3.5 (17)	2.0 (9)	23.7	14.2 (60)	9.5 (40)	1.7 (7)	4.7 (20)	3.0 (13)
55–59	17.7	11.6 (66)	6.0 (34)	0.8 (4)	3.2 (18)	2.0 (11)	20.0	11.2 (56)	8.8 (44)	1.4 (7)	4.4 (22)	3.0 (15)
60–64	14.6	9.0 (62)	5.6 (38)	0.6 (4)	2.9 (20)	2.0 (14)	16.5	8.5 (52)	8.0 (48)	1.1 (7)	3.9 (24)	2.9 (18)
65–69	11.8	6.8 (57)	5.0 (43)	0.5 (4)	2.6 (22)	2.0 (17)	13.2	6.3 (47)	7.0 (53)	0.9 (7)	3.3 (25)	2.8 (21)
70–74	9.3	4.9 (53)	4.3 (47)	0.4 (4)	2.1 (23)	1.8 (20)	10.4	4.5 (43)	5.9 (57)	0.7 (6)	2.7 (26)	2.5 (24)
75+	7.2	3.6 (50)	3.6 (50)	0.3 (4)	1.7 (24)	1.6 (23)	8.1	3.2 (40)	4.9 (60)	0.5 (6)	2.2 (27)	2.2 (27)

(%) = proportion of remaining life.

## Discussion

This study presents the application of health expectancies in describing the burden due to trachoma by dividing life expectancy into life spent without trichiasis and with trichiasis (trichiasis with normal vision, trichiasis with low vision and trichiasis with blindness). The methods can be applied to other trachoma endemic setting, and presents a technique of estimating the burden associated with uncontrolled trachoma. In Southern Sudan, life expectancy at birth for the year 2001 was 54.2 for men and 58.1 years for women. At age five years, men expected to live an eighth of remaining life with trichiasis and nearly a tenth of remaining life with visual impairment (low vision or blindness) due to trachoma. For women this rose to nearly a fifth of remaining life at age five with trichiasis and an eighth of remaining life with trachomatous visual impairment. Not only were women estimated to live longer, they were also expected to spend a greater amount of time with trichiasis, trichiasis with low vision, and trichiasis with blindness compared to men.

Health expectancy is a measure that combines information on both mortality and morbidity to derive lengths of time spent in different states of health. The Sullivan method for calculating health expectancies has advantages over previous summary measures of trachoma burden because it is presented in units of expected years of life with or without the disease condition. This is intuitively meaningful for policy makers and a non-technical audience, and compares favourably with other indicators such as mortality and incidence rates or disability adjusted life years (DALYs), which are not generally easily understood [24]. Other advantages of the method include simplicity and relative accuracy in addition to using data which are commonly available: prevalence data from surveys and life tables.

In common with other countries for which vital registration is not well established, the WHO life tables are the most authoritative for Southern Sudan. Until recently (April 2008), no population census has been undertaken for many years due to the civil war, and without any vital registration we acknowledge that the true picture for life expectancy in Southern Sudan is difficult to ascertain and may differ from the WHO estimates. Indeed, data collected during the war suggest a total life expectancy for both males and females of just 42 years [25]. However, the Modified Logit model life tables used by WHO were developed in order to address systematic deviations in mortality patterns observed as levels of child and adult mortality deviate from the standard, and this method has been used extensively by WHO to produce life tables for countries with poor vital registration [21].

Our study has a number of potential limitations. The random walk method, whilst acceptable for other purposes is not ideal where the outcome being assessed is one that is obvious to those involved in guiding the survey teams. Bias could have been introduced since the village guides may have been more likely to direct the survey teams to households where they knew there were persons with TT or visual impairment [26]. The Sullivan health expectancies are not appropriate for modelling dynamic changes associated with disease control interventions since it takes a long time for the age specific prevalence of a disability to reach the equilibrium values corresponding to the changes in age specific incidence rates [27]. In addition, cross-sectional data incorporate past recovery, incidence and death rates in the prevalence at particular ages; hence the effects of these rates on health expectancy are more difficult to disentangle [28]. Two other methods are used for calculating health expectancies: multiple-decrement life tables [29] and increment-decrement or multi-state life tables [30]. These methods employ longitudinal data and provide more robust basis for predicting service needs and may be useful in estimating the effects of trachoma control interventions, for instance, eyelid surgery for TT. However, unlike the Sullivan method, these later methods are less used due to lack of appropriate longitudinal data.

Data on distribution of vision status among persons with TT was only available for one district and these were applied to the population prevalence of TT calculated from a large sample from 11 districts, rather than modelling data for each district separately. However, a potential limitation with our VA models is that the effects of co-morbidity of other conditions leading to visual impairment such as refractive error were not controlled for in our model for distribution of VA [19]. Overall, there will be some people for whom this approach is likely to have resulted in an overestimate of trichiasis life expectancy. For others, this approach could have underestimated trichiasis life expectancy, since trachoma may have been causing a proportion of their vision loss, even if not the main cause of vision loss. The effects of co-morbidity thus operate in both directions and the overall bias in estimated potential gain in health expectancy is likely to be very small [31].

Consistent with other studies, our study showed longer life expectancy and trichiasis free life expectancy in females compared to males. In addition, females experienced greater proportions of years lived with trichiasis. Generally, the greater proportion of years lived with disability in females has been suggested to be as a result of longer overall survival or longer survival after the development of disability or disease [32]. Survey data from most trachoma endemic countries have consistently found the prevalence of scarring, trichiasis, and trachoma related blindness to be higher in females compared to males [33]. Therefore, the female excess in low vision or blindness associated with trichiasis is consistent with both greater survival and greater risk of trachomatous blindness among females.

### Conclusion

We have presented the burden of trachomatous vision loss by age and gender using health expectancies. These data are of value in advocacy for trachoma control in engagement with politicians and donors. Unless action is taken by further delivery of trachoma control interventions, then populations in Southern Sudan can expect to spend a substantial proportion of their life with low vision or blindness due to trachoma.
